# A Human Visual System Inspired No-Reference Image Quality Assessment Method Based on Local Feature Descriptors

**DOI:** 10.3390/s22186775

**Published:** 2022-09-07

**Authors:** Domonkos Varga

**Affiliations:** Ronin Institute, Montclair, NJ 07043, USA; domonkos.varga@ronininstitute.org

**Keywords:** no-reference image quality assessment, quality-aware features, keypoint detector

## Abstract

Objective quality assessment of natural images plays a key role in many fields related to imaging and sensor technology. Thus, this paper intends to introduce an innovative quality-aware feature extraction method for no-reference image quality assessment (NR-IQA). To be more specific, a various sequence of HVS inspired filters were applied to the color channels of an input image to enhance those statistical regularities in the image to which the human visual system is sensitive. From the obtained feature maps, the statistics of a wide range of local feature descriptors were extracted to compile quality-aware features since they treat images from the human visual system’s point of view. To prove the efficiency of the proposed method, it was compared to 16 state-of-the-art NR-IQA techniques on five large benchmark databases, i.e., CLIVE, KonIQ-10k, SPAQ, TID2013, and KADID-10k. It was demonstrated that the proposed method is superior to the state-of-the-art in terms of three different performance indices.

## 1. Introduction

With the continuous development of imaging systems, the demand for innovative, objective image quality assessment (IQA) methods is growing. Since digital images are subjects of a variety of distortions and noise types during image acquisition [1], compression [2], reconstruction [3], and enhancement [4], image quality assessment has also many practical applications [5] in medical imaging [6], remote sensing imaging [7], monitoring the quality of streaming applications [8], or benchmarking image processing algorithms [9] under different distortions. Thus, objective IQA has been a subject of intensive research in the image processing community to replace subjective quality evaluation of digital images which is a time-consuming, expensive, and laborious process [10].

In the literature, objective IQA measures are traditionally divided into three branches [11], such as full-reference (FR), reduced-reference (RR), and no-reference (NR) IQA, with respect to the availability of the distortion-free—very often referred as reference—images. As the terminology implies, FR techniques evaluate the perceptual quality of a distorted image with full access to its reference image, while NR algorithms cannot rely on reference images. For RR methods, partial information about the reference images is available.

### 1.1. Contributions

The contributions of this work are as follows. Although the deep learning paradigm dominates the field of objective IQA [12,13], the interest in methods which simulates the sensitivity of HVS to statistical regularities and structures is also a hot research topic in the literature [14,15]. In our previous work [16], it was empirically corroborated that the statistics of local feature descriptors are quality-aware features. Here, we use systematically the statistics of local feature descriptors to compile a powerful feature vector for NR-IQA. Specifically, multiple HVS inspired filters are applied to the color channels of an input image to generate feature maps where the HVS sensitive statistical regularities are emphasized. Next, the statistics of local feature descriptors, such as KAZE [17] or BRISK [18], are extracted from the feature maps as quality-aware features. Since there is a close connection between human visual perception and low-level visual features [19,20], various statistics of local feature descriptors is able to provide a powerful feature representation for NR-IQA. The effectiveness of the proposed method is empirically corroborated in tests recommended by the IQA community using publicly available large IQA benchmark databases, i.e., CLIVE [21], KonIQ-10k [22], SPAQ [23], TID2013 [24], and KADID-10k [25].

### 1.2. Structure of the Paper

The rest of this study is organized as follows. In Section 2, previous and related work are summarized. Section 3.1 provides an overview about the applied benchmark databases, gives the definition of the evaluation metrics, and defines the evaluation protocol. In Section 3.2, we introduce our proposed method. Section 4 presents the experimental results and a comparison to the state-of-the-art from various aspects. Finally, a conclusion is drawn in Section 5.

## 2. Related Work

NR-IQA algorithms can be classified into learning-free and learning-based categories. As the name indicates, learning-based methods rely on various machine and/or deep learning techniques to construct a model for perceptual quality estimation. Learning-free methods can be further divided into two groups, i.e., spatial [26] and spectral domain [27] based approaches. A common method [28] for score prediction involves fitting a portion of the training data to the joint distribution of the feature vector and the related opinion scores. Given the test data feature vector, the score prediction in this instance entails maximizing the likelihood of the test data opinion score. Other methods [29] that are both opinion- and distortion-unaware measure the separation in sparse feature space between the reference and distorted images. In contrast, Leonardi et al. [30] elaborated an opinion-unaware method that exploits the activation maps of pretrained convolutional neural networks [31] by considering the correlations between feature maps.

Classical machine learning based algorithms utilized for choice natural scene statistics (NSS) [32] and support vector regressors (SVR) [33]. According to our understanding, the evolution of the human visual system (HVS) has been driven by natural selection. Therefore, HVS assimilated a comprehensive knowledge about the regularity of our natural environment. In addition, researchers have pointed out [34] that certain image structure regularities deteriorate in the presence of noise and the deviation from them can be exploited for image quality evaluation. Classical methods utilizing NSS include for example BLIINDS-II [35], BRISQUE [36], CurveletQA [37], and DIIVINE [38]. Specifically, Saad et al. [35] constructed an NSS model from discrete cosine transform (DCT) coefficients of an image and fitted generalized Gaussian distributions on the coefficients to obtain their shape parameters which were used as quality-aware attributes and mapped onto quality scores with a trained SVR. In contrast, Jenadeleh and Moghaddam [39] proposed a Wakeby distribution statistical model to extract quality-aware features. Moorthy et al. [38] utilized steerable pyramid decomposition [40]—an overcomplete wavelet transform—using across multiple orientations and scales. In contrast, Mittal et al. [36] applied the spatial domain for NSS model construction. To be more specific, quality-aware features were derived from locally normalized luminance coefficients and mapped onto perceptual quality with a trained SVR. Liu et al. [37] proposed a two-stage framework incorporating a distortion classification and a quality prediction step. Further, quality-aware features were derived from the curvelet representation of the input image. Specifically, the authors emprirically proved that the coordinates of the maxima given in log-histograms of the curvelet coefficients, the energy distributions, and the scale are good predictors of image perceptual quality. Based on the observation that image distortions can significantly modify the shapes of objects present in the image, Bagade et al. [41] introduced shape adaptive wavelet features for NR-IQA. In contrast, Jenadeleh et al. [42] boosted already existing NR-IQA features with features proposed for image aesthetics assessment. Further, they demonstrated that aesthetic aware features are able to increase the performance of perceptual quality estimation.

Recently, deep neural networks, particularly convolutional neural networks (CNN) have gained a significant amount of attention in the literature due to their improved performance in many fields [43,44,45,46] compared to other approaches and paradigms. In NR-IQA, Kang et al. [47] applied first a CNN successfully. Namely, the authors implemented a traditional CNN which accepts image patches of 32×32 and predicts the patches’ quality independently from each other. The entire image’s perceptual quality was obtained by taking the arithmetic mean of the patches’ quality scores. Similar to [47], Kim and Lee [48] trained a CNN on image patches but the patches’ desired quality scores were determined by a traditional FR-IQA metric which restricts this method to the evaluation of artificially distorted images. Bare et al. [49] developed a network that operates on image patches similar to [47] but the patches target score is calculated from a traditional FR-IQA metric (feature similarity index [50]) similar to [48]. On the whole, the entire image’s perceptual quality is estimated by the predicted feature similarity index [50] scores of the image patches. In contrast, Conde et al. [51] took a CNN backbone network and trained it using a loss function [52] which aims to minimize the mean squared error and maximize linear correlation coefficient between the predicted and ground-truth quality scores. Further, the authors applied several data augmentation techniques, such as horizontal flips, vertical flips, rotations, and random cropping. To handle images with different aspect ratios, Ke et al. [53] introduced a transformer [54] based NR-IQA model which applied a hash-based 2D absolute-position-encoding for embedding image patches extracted from multiple scales. In contrast, Zhu et al. [55] embedded the input images’ original aspect ratios into the self-attention module of a swin transformer [56]. Sun et al. [57] introduced the distortion graph representation framework which contains a distortion type discrimination network aiming to discriminate between distortion types and a fuzzy prediction network for perceptual quality estimation. Liu et al. [58] introduced lifelong learning for NR-IQA to learn new distortion types without accessing to previous training data. First, the authors utilized a split-and-merge distillation strategy for compiling a single-head regression network. In the split phase, a distortion-specific generator was implemented for generating pseudo-features for unseen distortions. In the merge phase, these pseudo-features were coupled with pseudo-labels to distill knowledge about distortions.

A general, in-depth overview about the field of NR-IQA is out of the scope of this paper. For more details, we refer to the PhD thesis of Jenadeleh [59] and the book of Xu et al. [60] Besides natural images, there are other modalities, whose no-reference quality assessment are also investigated in the literature, such as stereoscopic images [61], light field images [62], or virtual reality [63].

## 3. Materials and Methods

### 3.1. Materials

In this part of the paper, the applied IQA benchmark databases and the evaluation protocol are discussed in detail.

#### 3.1.1. Applied IQA Benchmark Databases

**Figure 1:**
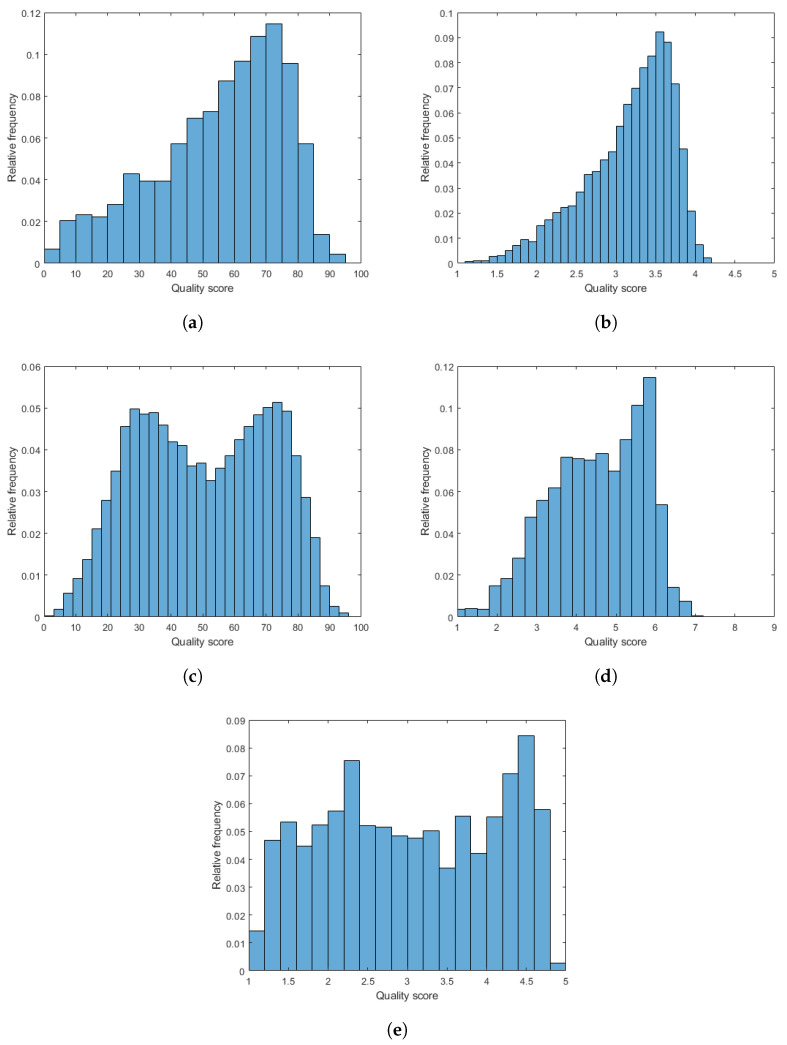
The empirical distributions of quality scores in the applied IQA databases. (**a**) CLIVE [21], (**b**) KonIQ-10k [22], (**c**) SPAQ [23], (**d**) TID2013 [24], (**e**) KADID-10k [25].

In this paper, we applied five publicly available IQA benchmark, such as CLIVE [21], KonIQ-10k [22], SPAQ [23], TID2013 [24], and KADID-10k [25], to evaluate and compare our proposed methods to the state-of-the-art. Specifically, CLIVE [21], KonIQ-10k [22], and SPAQ [23] contain unique quality labeled images with authentic distortions. The quality ratings were collected in a crowdsourcing experiment [64,65,66] for CLIVE [21] and KonIQ-10k [22], while the quality ratings were obtained in a traditional laboratory environment for SPAQ [23]. Further, CLIVE [21] and KonIQ-10k [22] contain images with fixed resolution. On the other hand, there is no fixed resolution in SPAQ [23] but the images have high resolution which varies around 4000×4000. In contrast to CLIVE [21], KonIQ-10k [22], and SPAQ [23], TID2013 [24] and KADID-10k [25] consist of 24 and 25 reference images whose perceptual quality are considered perfect, respectively. The quality labeled distorted images were produced artificially by an image processing tool from the reference images using different distortion types (i.e., JPEG compression noise, salt & pepper noise, Gaussian blur, etc.) at multiple distortion levels. The main properties of the applied IQA benchmark databases are summarized in Table 1. Further, the empirical distributions of quality scores are depicted in Figure 1.

#### 3.1.2. Evaluation Protocol and Metrics

The assessment of NR-IQA algorithms involves the measurement of the correlation strength between the predicted scores and the ground-truth scores of an IQA benchmark database. As common in the literature, about 80% of images was used for training and the remaining 20% was used for testing in our experiments. Further, databases with artificial distortions were divided into training and test sets with respect to the reference images to prevent semantic content overlap between these two sets.

In this paper, the medians of Pearson linear correlation coefficients (PLCC), Spearman rank order correlation coefficient (SROCC), and Kendall rank order correlation coefficient (KROCC), which were measured over 100 random train-test splits, are given to characterize the performance of the proposed method and other examined state-of-the-art methods. However, there is a non-linear relationship between the predicted and the ground-truth scores. This is why, a non-linear logistic regression was applied before the computation of PLCC as advised by [67]:

(1) $$Q_{f}=\beta_{1}\left(\frac{1}{2}-\frac{1}{1+\exp(\beta_{2}\left(Q_{p}-\beta_{3}\right))}\right)+\beta_{4}Q_{p}+\beta_{5},$$

where $Q_{f}$ and $Q_{p}$ stand for the fitted and predicted score, respectively. Further, the regression parameters are denoted by $\beta_{i}$’s (i=1,…,5).

### 3.2. Proposed Method

**Figure 2:**
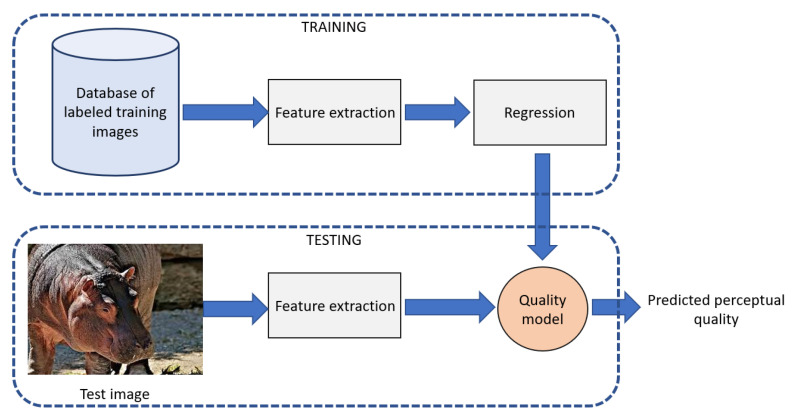
High-level overview of the proposed NR-IQA algorithm.

The high-level overview of the proposed NR-IQA algorithm is depicted in Figure 2. It can be observed that the proposed method is built upon two distinct steps. In the first, training step, local features are extracted from a database of quality labeled, training images. Next, a regression model is trained based on them to obtain a quality model. This model is used to estimate the perceptual quality of a previously unseen image in the testing step.

**Figure 3:**
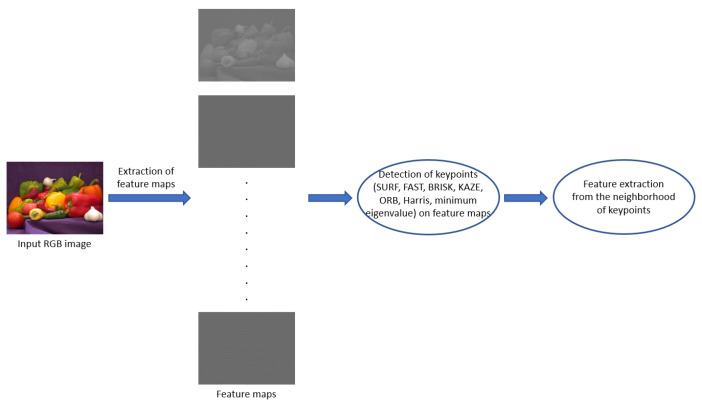
High-level overview of local feature extraction.

Image distortions influence the human visual system’s (HVS) sensitivity to local image structures, such as edges or texture elements [68]. For that reason, many NR-IQA methods have been proposed [69,70,71] in the literature to compile a quality model from them. However, edge information or local binary patterns are not always able to provide powerful feature representation for NR-IQA. Therefore, in this study, an application of local feature descriptors and HVS-inspired filters are investigated thoroughly to extract quality-aware local features and compile a powerful feature representation for NR-IQA. Figure 3 depicts the general process of local quality-aware feature extraction. First, the input RGB image is filtered by a set of HVS inspired filters to create feature maps. On these maps, local keypoints are detected by local feature descriptors (such as SURF [72]). Finally, feature extraction is carried out from the neighborhoods of the detected keypoints.

In the proposed method, an input RGB image is converted into YCbCr color space, since the chroma component is separated from the color information in YCbCr. The conversion from RGB to YCbCr was carried out using the following equation [73]:

(2) $$\left(\begin{array}{c} Y\\ Cb\\ Cr \end{array}\right)=\left(\begin{array}{ccc} 0.2568 & 0.5041 & 0.0979\\ -0.1482 & -0.2910 & 0.4392\\ 0.4392 & -0.3678 & -0.0714 \end{array}\right)\left(\begin{array}{c} R\\ G\\ B \end{array}\right),$$

where *R*, *G*, and *B* stand for the red, green, and blue color channels, respectively. Subsequently, a color channel $C_{i}$ is filtered using different HVS-inspired filters to obtain multiple feature maps. Since local feature descriptors treat images from the HVS’s point of view, their statistics are able to provide quality-aware features [16]. Further, the applied HVS inspired filters emphasize those statistical regularities of a natural scene which are highly sensitive to image distortions from the perspective of HVS. Specifically, 5 statistical features are derived from each filtered color channels using the statistics of different local feature descriptors. In Section 3.3, Section 3.4 and Section 3.5, the compilation of HVS inspired feature maps are described. Next, the proposed quality-aware feature extraction from the feature maps is described in Section 3.6.

### 3.3. Bilaplacian Feature Maps

First, Bilaplacian feature maps were obtained using Bilaplacian filters. In [74], Gerhard et al. demonstrated that the HVS is highly adapted to statistical regularities of images. Further, zero-crossings [75] in an image occur where the gradient starts increasing or decreasing and help the HVS in interpreting the image. Using the idea of zero-crossings, Ghosh et al. [76] pointed out that the behaviour of the extended classical receptive field of retinal ganglion cells can be modeled as a combination of three zero-mean Gaussians at three different scales which are equivalent to are the Bilaplacian of the Gaussian filter [77]. The L(x,y) Laplacian of an I(x,y) image can be expressed as:

(3) $$L\left(x,y\right)=\frac{\partial^{2}I\left(x,y\right)}{\partial x^{2}}+\frac{\partial^{2}I\left(x,y\right)}{\partial y^{2}}.$$

Since a digital image is represented as a set of discrete pixels, discrete convolution kernels are used to approximate the Laplacian. In this paper, the following kernels are considered:

(4) $$L_{1}=\left(\begin{array}{ccc} 0 & 1 & 0\\ 1 & -4 & 1\\ 0 & 1 & 0 \end{array}\right),L_{2}=\left(\begin{array}{ccc} 1 & -2 & 1\\ -2 & 4 & -2\\ 1 & -2 & 1 \end{array}\right),L_{3}=\left(\begin{array}{ccc} 1 & 0 & 1\\ 0 & -4 & 0\\ 1 & 0 & 1 \end{array}\right),$$

(5) $$L_{4}=\left(\begin{array}{ccc} -2 & 1 & -2\\ 1 & 4 & 1\\ -2 & 1 & -2 \end{array}\right),L_{5}=\left(\begin{array}{ccc} -1 & -1 & -1\\ -1 & 8 & -1\\ -1 & -1 & -1 \end{array}\right).$$

Bilaplacian kernels are obtained by convolving two Laplacian kernels:

(6) $$L_{ij}^{2}=L_{i}*L_{j},$$

where * stands for the convolution operator. In our study, $L_{11}^{2}$, $L_{22}^{2}$, $L_{33}^{2}$, $L_{44}^{2}$, $L_{55}^{2}$, $L_{13}^{2}$, and $L_{24}^{2}$ Bilaplacian kernels were considered. Subsequently, a set of feature maps is derived from the input image by filtering with the Bilaplacian kernels the *Y*, Cb, and Cr channels. To be more specific, 3×7=21 feature maps are obtained by filtering 3 color channels (*Y*, Cb, Cr) with 7 filters ( $L_{11}^{2}$, $L_{22}^{2}$, $L_{33}^{2}$, $L_{44}^{2}$, $L_{55}^{2}$, $L_{13}^{2}$, $L_{24}^{2}$).

### 3.4. High-Boost Feature Maps

High-boost filtering is used to enhance high-frequency image regions which the HVS is also sensitive for [78]. Similarly to the previous subsection, high-boost filtering is applied on the color channels of *Y*, Cb, and Cr to strengthen high-frequncy information. In this case, the convolution kernel is the following:

(7) $$H=\left(\begin{array}{ccc} -C & -C & -C\\ -C & 8C+1 & -C\\ -C & -C & -C \end{array}\right),$$

where *C* is a constant value which controls the enhancement difference between a pixel location and its neighborhood. In our study, C=1 was used. However, image distortions can occur at different scales. Therefore, a color channel was filtered 4 times in succession to obtain four feature maps from one channel. Since we have three channels, 3×4=12 feature maps were extracted in total applying high-boost filtering.

### 3.5. Derivative Feature Maps

In [74], Gerhard et al. draw the inference that the HVS is biased for processing natural images. Further, it has a large knowledge of statistical regularities in images. In [79], Li et al. demonstrated that derivatives and higher order derivatives are related to different statistical regularities of a natural scene. Therefore, there are good features for NR-IQA. For instance, higher order derivatives may be able to capture detailed discriminative information, while first order derivative information is typically related to the slope and elasticity of a surface. Second order derivatives intended to capture the geometric qualities associated to curvature [80]. Motivated by these previous works, we used the following convolution of two derivative kernels to filter *Y*, Cb, Cr color channels of an input image:

(8) $$D_{1}=\left(\begin{array}{ccc} -1 & 1 & -1\\ 1 & 0 & 1\\ -1 & 1 & -1 \end{array}\right)*\left(\begin{array}{ccc} 1 & -1 & 1\\ -1 & 0 & -1\\ 1 & -1 & 1 \end{array}\right).$$

Similarly, we can define $D_{2}$, $D_{3}$, $D_{4}$, and $D_{5}$ masks for 5×5, 7×7, 11×11, and 13×13 sizes. Finally, the derivative feature maps are obtained by filtering *Y*, Cb, Cr color channels with $D_{1}$, $D_{2}$, $D_{3}$, $D_{4}$, and $D_{5}$. As a result, 3×5=15 derivative feature maps were obtained.

### 3.6. Feature Extraction

As one can see from the previous subsections, 21 Bilaplacian feature maps, 12 high-boost feature maps, and 15 derivative feature maps were generated which means in total 21+12+15=48 feature maps. In the feature extraction step, 7×N keypoints are detected using 7 different local keypoint detectors, i.e., SURF (speed up robust features) [72], FAST (features from accelerated segment test) [81], BRISK (binary robust invariant scalable keypoints) [18], KAZE (Japanese word that means wind) [17], ORB (oriented FAST and rotated binary robust independent elementary features) [82], Harris [83], and minimum eigenvalue [84], in each feature map. For each keypoint, its M×M rectangular neighborhood with the keypoint’s location as center point is taken. Further, each M×M rectangular block in each Bilaplacian, high-boost, and derivative feature maps are characterized by the mean, median, standard deviation, skewness, and kurtosis of the grayscale values found in the involved block. The skewness of a set of *n* elements is determined as

(9) $$s=\frac{\frac{1}{n}\sum_{i=1}^{n}{\left(x_{i}-\bar{x}\right)}^{3}}{{\left(\sqrt{\frac{1}{n}\sum_{i=1}^{n}{\left(x_{i}-\bar{x}\right)}^{2}}\right)}^{2}},$$

where $\bar{x}$ is the arithmetic mean of all $x_{i}$ elements. Similarly, the kurtosis can be given as

(10) $$k=\frac{\frac{1}{n}\sum_{i=1}^{n}{\left(x_{i}-\bar{x}\right)}^{4}}{{\left(\frac{1}{n}\sum_{i=1}^{n}{\left(x_{i}-\bar{x}\right)}^{2}\right)}^{3}}.$$

Using the statistics of the rectangular blocks, a given feature map is characterized by the arithmetic mean of all blocks’ statistics. As a result, a 3×7×7×5=735 dimensional feature vector is obtained using Bilaplacian filters and the statistics of local feature descriptors, since 3 color channels were filtered with 7 Bilaplacian kernels and 7 different feature descriptors with 5 different statistics were applied. Further, a 3×4×7×5=420 dimensional feature vector is obtained using the high-boost filters, since 3 color channels were filtered with 4 high-boost kernels and 7 different feature descriptors with 5 different statistics were applied as in the previous case. Similarly, 3×5×7×5=525 dimensional feature vector is obtained from the derivative feature maps. By concatenating the fore-mentioned vectors, a 735+420+525=1680 dimensional feature vector can be derived which can be mapped onto perceptual quality scores with a machine learning technique.

**Figure 4:**
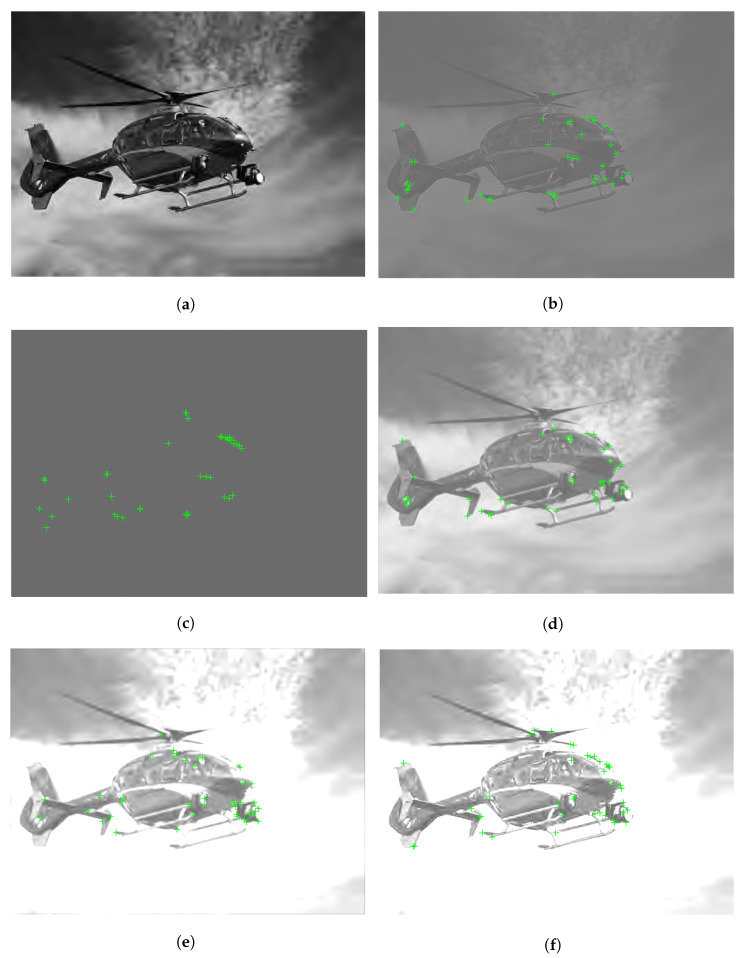

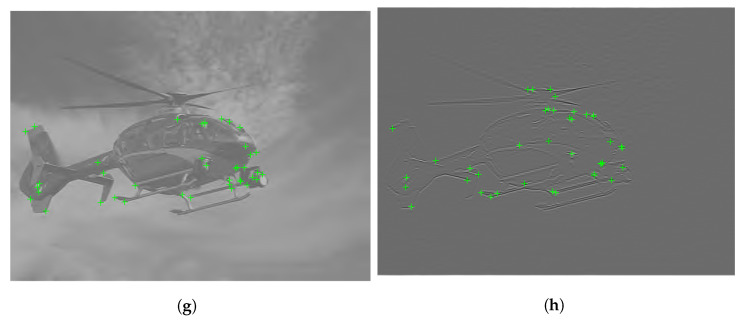
Illustration of Bilaplacian feature maps and detected FAST keypoints. (**a**) **Y** channel, (**b**) $Y*L_{11}^{2}$, (**c**) $Y*L_{22}^{2}$, (**d**) $Y*L_{33}^{2}$, (**e**) $Y*L_{44}^{2}$, (**f**) $Y*L_{55}^{2}$, (**g**) $Y*L_{13}^{2}$, (**h**) $Y*L_{24}^{2}$.

**Figure 5:**
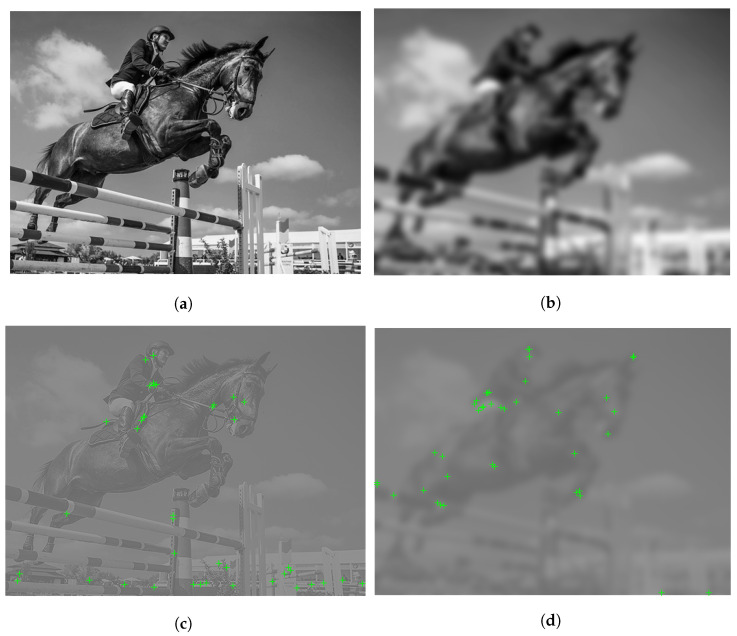
Illustration of Bilaplacian feature map and the detected FAST keypoints on two different distortion levels. (**a**) Luminance channel of pristine image $\left(Y_{pristine}\right)$, (**b**) Luminance channel of distorted image $\left(Y_{distorted}\right)$, (**c**) $Y_{pristine}*L_{11}^{2}$, (**d**) $Y_{distorted}*L_{11}^{2}$.

As an illustration, Figure 4 depicts a Y channel and its Bilaplacian feature maps with the detected FAST keypoints. From this illustration it can be seen that keypoints are accumulated around those regions which highly influence humans’ quality perception. In Figure 5, it is illustrated that the location of keypoints on the feature maps is changing with respect to the strength of image distortion. As a consequence, it seems justified that the statistics of local feature descriptors on carefully chosen feature maps are quality-aware features.

### 3.7. Perceptual Quality Estimation

The quality model formally can be written as: q=G(F), where q is a vector of quality scores, F is a set of extracted feature vectors, and *G* denotes the quality model. Specifically, *G* can be determined by a properly chosen machine learning (regression) technique. In this study, we made experiments with two different regression methods, i.e., support vector regressor (SVR) and Gaussian process regressor (GPR). In the followings, we denote the proposed methods by LFD-IQA-SVR and LFD-IQA-GPR with respect to the applied regression method. In the chosen codename, LFD refers to the abbreviation of local feature descriptors whose statistics were utilized as quality-aware features.

## 4. Experimental Results

In this section, our numerical experimental results are presented. First, an ablation study is carried out in Section 4.1 first to justify certain design choices of the proposed methods. In the following subsection, a comparison to several other state-of-the-art methods is presented using accepted publicly available benchmark databases and evaluation protocol described in Section 3.1. This comparison involves direct and cross-database tests as well as significance tests.

### 4.1. Ablation Study

**Figure 6:**
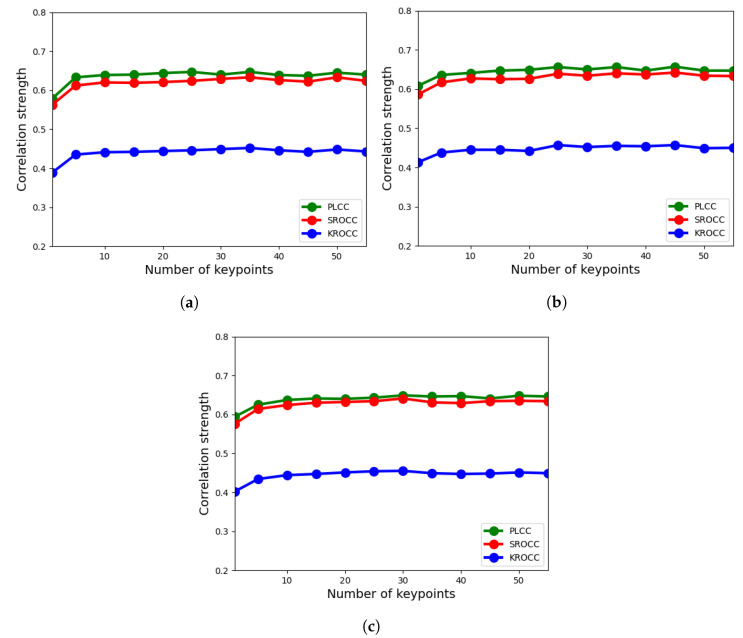
Ablation study for LFD-IQA-SVR: number of detected keypoints vs. correlation strength. (**a**) 3×3 block size, (**b**) 5×5 block size, (**c**) 7×7 block size.

**Figure 7:**
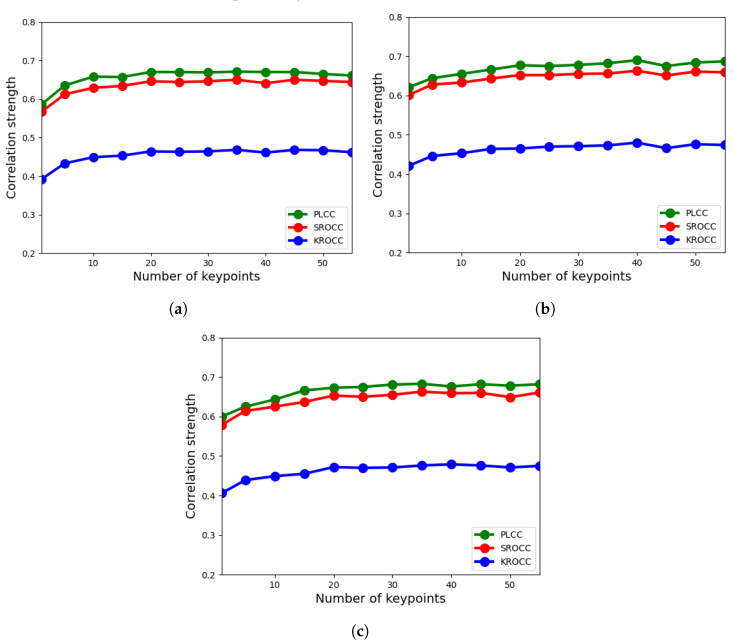
Ablation study for LFD-IQA-GPR: number of detected keypoints vs. correlation strength. (**a**) 3×3 block size, (**b**) 5×5 block size, (**c**) 7×7 block size.

In the proposed feature extraction methodology, there are two tunable parameters, i.e., the *N* number of detected keypoints for each feature feature descriptor and the M×M block size. In this ablation study, CLIVE [21] was utilized using the evaluation protocol given in Section 3.1 to determine an optimal value for these two parameters. To be more specific, we varied the number of detected keypoints from 1 to 55 and we experimented with 3 different block sizes, i.e., 3×3, 5×5, and 7×7. The results for LFD-IQA-SVR and LFD-IQA-GPR are summarized in Figure 6 and Figure 7, respectively. From these results, it can be seen that 5×5-sized neighborhood is the optimal choice for both LFD-IQA-SVR and LFD-IQA-GPR. On the other hand, LFD-IQA-SVR achieves its best performance at 45 detected keypoints while LFD-IQA-GPR has its peak performance at 40 detected keypoints. Therefore, we applied 5×5 neighborhoods and 45 or 40 keypoints, respectively.

The proposed methods were implemented and tested in MATLAB R2022a. To be more specific, the Computer Vision Toolbox’s functions were utilized for the detection of keypoints and feature extraction, while the Statistics and Machine Learning Toolbox was used in the regression part of the proposed method.

### 4.2. Comparison to the State-of-the-Art

**Figure 8:**
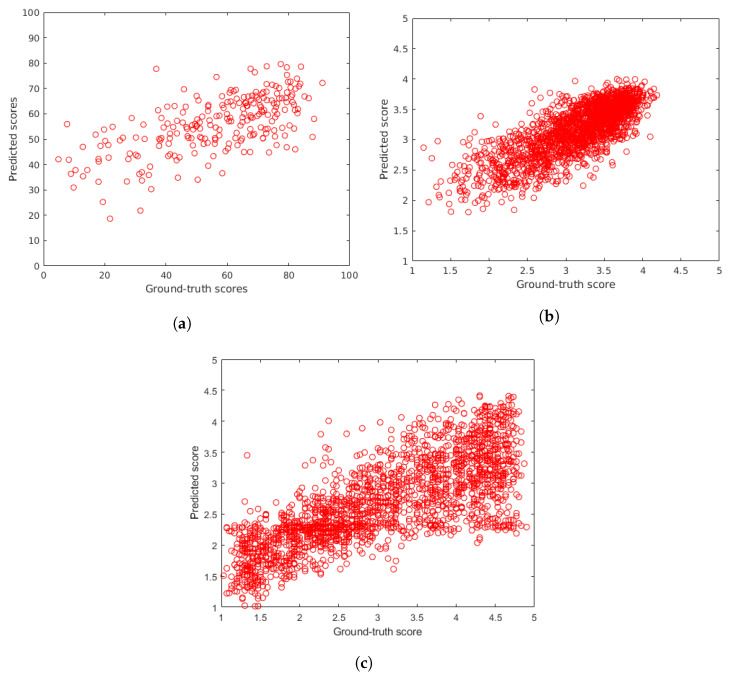
Ground-truth scores versus predicted scores using the proposed LFD-IQA-SVR method on (**a**) CLIVE [21], (**b**) KonIQ-10k [22], and (**c**) KADID-10k [25] test sets.

**Figure 9:**
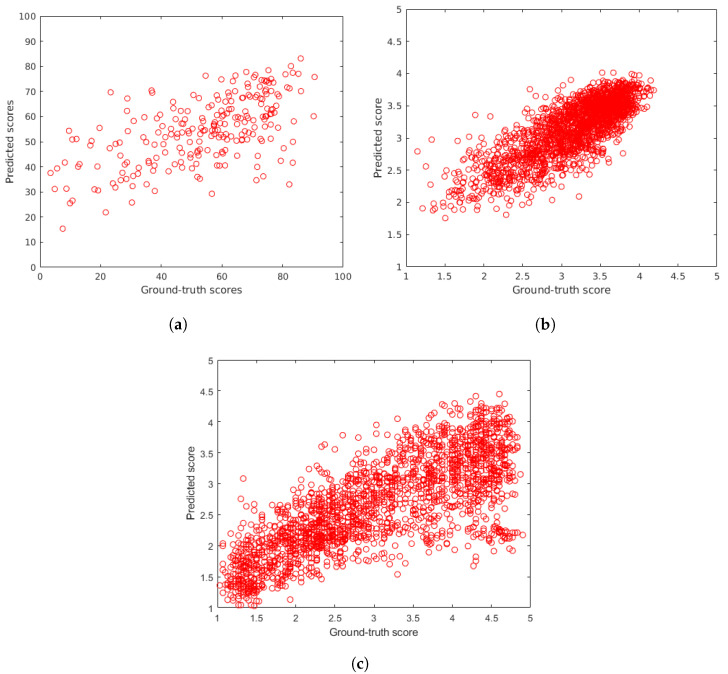
Ground-truth scores versus predicted scores using the proposed LFD-IQA-GPR method on (**a**) CLIVE [21], (**b**) KonIQ-10k [22], and (**c**) KADID-10k [25] test sets.

The proposed methods were compared to the following 16 state-of-the-art methods: BIQI [85], BLIINDS-II [35], BMPRI [86], BRISQUE [36], CurveletQA [37], DIIVINE [38], ENIQA [87], GRAD-LOG-CP [69], GWH-GLBP [70], IL-NIQE [27], NBIQA [88], NIQE [26], OG-IQA [71], PIQE [89], Robust BRISQUE [90], and SSEQ [91]. Excluding the training-free IL-NIQE [27], NIQE [26] and, PIQE [89], these methods were evaluated as the same way as the proposed methods. To provide a fair comparison, the same subsets of images were selected in the random 100 train-test splits. Since IL-NIQE [27], NIQE [26] and, PIQE [89] are opinion unaware methods, they were tested on the applied benchmark databases in one iteration measuring PLCC, SROCC, and KROCC on the entire database without any train-test splits. In Table 2 and Table 3, the median values measured over 100 random train-test splits for the considered and proposed NR-IQA methods on authentic distortions (CLIVE [21], KonIQ-10k [22], SPAQ [23]) are reported. Similarly, Table 4 summarizes the results on artificial distortions. From these results, it can be seen that the proposed LFD-IQA-SVR achieves the second best results in almost all cases, while the proposed LFD-IQA-GPR provides the best results for all databases in all performance metrics. In Table 5, the results measured on the individual databases are aggregated into direct and weighted averages of PLCC, SROCC, and KROCC. From these results, it can be concluded that the proposed methods are able to outperform all the other methods by a large margin. Further, the difference between the proposed and the other algorithms is larger in case of weighted averages. This indicates that the proposed methods tend to give a better performance on larger IQA databases. Figure 8 and Figure 9 depict ground-truth versus predicted quality score scatter plots of the proposed methods determined on CLIVE [21], KonIQ-10k [22], and KADID-10k [25] test sets, respectively.

To prove that achieved results summarized in Table 2, Table 3 and Table 4 are significant, the Wilcoxon rank sum test was applied [69,92]. To be specific, the null hypothesis was that two sets of 100 SROCC values produced by two different NR-IQA methods were sampled from continuous distributions with equal median values. In our tests, 5% significance level was applied. The results are summarized in Table 6 for LFD-IQA-SVR, while the results are shown in Table 7 for LFD-IQA-GPR. Here, symbol ’1’ is used to denote that the proposed method is significantly better than the method in the row on the database in the column. From the presented results, it can be clearly seen that the achieved result is significant compared to the state-of-the-art. As a consequence, the proposed HVS-inspired feature extraction method have proved to be more effective than the those of the examined state-of-the-art methods.

In an other test, the generalization ability of the methods were examined. Namely, the algorithms were trained on the entire KonIQ-10k [22] database used as a training set and tested on the entire CLIVE [21] used as a test set. This process is called cross database test in the literature [93]. The results of the cross database are shown in Table 8. In this test, the proposed methods are also the best performing ones. Namely, they are able to outperform the state-of-the-art by a large margin.

## 5. Conclusions

In this paper, a novel machine learning based NR-IQA method was introduced which applies an innovative quality-aware feature extraction procedure relying on the statistics of local feature descriptors. To be more specific, a sequence of HVS inspired filters were applied to *Y*, Cb, and Cr color channels of an input image to enhance those statistical regularities of the image to which the HVS is sensitive. Next, certain statistics of various local feature descriptors were extracted from each feature map to construct a powerful feature vector which is able to characterize possible image distortions from various points of view. Finally, the obtained feature vector is mapped onto perceptual quality scores with a trained regressor. The proposed method was compared to 16 state-of-the-art NR-IQA methods on five large benchmark IQA databases containing either authentic (CLIVE [21], KonIQ-10k [22], SPAQ [23]) or artificial (TID2013 [24], KADID-10k [25]) distortions. Specifically, the comparison involved the demonstration of three performance metrics on direct database tests, significance tests, and a cross database test. As shown, the proposed method is able to outperform significantly the state-of-the-art and provides competitive results. Future work involves a real-time GPU (graphical processing unit) implementation of the proposed method. Another direction of future research is to generalize the achieved results to other types of image modalities, such as stereoscopic or computer-generated images. 

## Figures and Tables

**Table 1:** The main characteristics of the applied IQA benchmark databases.

Attribute	CLIVE [21]	KonIQ-10k [22]	SPAQ [23]	TID2013 [24]	KADID-10k [25]
#Distorted images	1162	10,073	11,125	3000	10,125
#Reference images	-	-	-	25	81
#Distortion types	-	-	-	24	25
#Distortion levels	-	-	-	5	5
Resolution	500×500	1024×768	∼4000×4000	512×384	512×384
#Subjects	8100	1467	600	971	2209
#Annotations	1400	1,200,000	186,400	27,000	303,750
Scale of quality scores	0–100	1–5	0–100	0–9	1–5
Subjective methodology	crowdsourcing	crowdsourcing	laboratory	laboratory	crowdsourcing
Year	2017	2018	2020	2013	2019

**Table 2:** Comparison to the state-of-the-art on CLIVE [21] and KonIQ-10k [22] databases. Median PLCC, SROCC, and KROCC values were measured over 100 random train-test splits. The best results are typed in bold, the second best results are underlined, and the third best results are typed in italic.

	CLIVE [21]			KonIQ-10k [22]		
**Method**	**PLCC**	**SROCC**	**KROCC**	**PLCC**	**SROCC**	**KROCC**
BIQI [85]	0.519	0.488	0.329	0.688	0.662	0.471
BLIINDS-II [35]	0.473	0.442	0.291	0.574	0.575	0.414
BMPRI [86]	0.541	0.487	0.333	0.637	0.619	0.421
BRISQUE [36]	0.524	0.497	0.345	0.707	0.677	0.494
CurveletQA [37]	*0.636*	*0.621*	0.421	0.730	0.718	0.495
DIIVINE [38]	0.617	0.580	0.405	0.709	0.693	0.471
ENIQA [87]	0.596	0.564	0.376	0.761	0.745	*0.544*
GRAD-LOG-CP [69]	0.607	0.604	0.383	0.705	0.696	0.501
GWH-GLBP [70]	0.584	0.559	0.395	0.723	0.698	0.507
IL-NIQE [27]	0.487	0.415	0.280	0.463	0.447	0.306
NBIQA [88]	0.629	0.604	*0.427*	*0.771*	*0.749*	0.515
NIQE [26]	0.328	0.299	0.200	0.319	0.400	0.272
OG-IQA [71]	0.545	0.505	0.364	0.652	0.635	0.447
PIQE [89]	0.172	0.108	0.081	0.208	0.246	0.172
Robust BRISQUE [90]	0.522	0.484	0.330	0.718	0.668	0.477
SSEQ [91]	0.487	0.436	0.309	0.589	0.572	0.423
LFD-IQA-SVR	0.669	0.647	0.464	0.786	0.769	0.569
LFD-IQA-GPR	**0.696**	**0.667**	**0.480**	**0.801**	**0.775**	**0.577**

**Table 3:** Comparison to the state-of-the-art on SPAQ [23] database. Median PLCC, SROCC, and KROCC values were measured over 100 random train-test splits. The best results are typed in bold, the second best results are underlined, and the third best results are typed in italic.

Method	PLCC	SROCC	KROCC
BIQI [85]	0.783	0.776	0.566
BLIINDS-II [35]	0.676	0.675	0.486
BMPRI [86]	0.739	0.734	0.506
BRISQUE [36]	0.726	0.720	0.518
CurveletQA [37]	0.793	0.774	0.503
DIIVINE [38]	0.774	0.756	0.514
ENIQA [87]	*0.813*	*0.804*	*0.603*
GRAD-LOG-CP [69]	0.786	0.782	0.572
GWH-GLBP [70]	0.801	0.796	0.542
IL-NIQE [27]	0.374	0.348	0.297
NBIQA [88]	0.802	0.793	0.539
NIQE [26]	0.264	0.310	0.206
OG-IQA [71]	0.726	0.724	0.594
PIQE [89]	0.211	0.156	0.091
Robust BRISQUE [90]	0.735	0.731	0.524
SSEQ [91]	0.745	0.742	0.549
LFD-IQA-SVR	0.852	0.847	0.627
LFD-IQA-GPR	**0.869**	**0.864**	**0.664**

**Table 4:** Comparison to the state-of-the-art on TID2013 [24] and KADID-10k [25] databases. Median PLCC, SROCC, and KROCC values were measured over 100 random train-test splits carried out with respect to the reference images. The best results are typed in bold, the second best results are underlined, and the third best results are typed in italic.

	TID2013 [24]			KADID-10k [25]		
**Method**	**PLCC**	**SROCC**	**KROCC**	**PLCC**	**SROCC**	**KROCC**
BIQI [85]	0.468	0.296	0.207	0.302	0.294	0.206
BLIINDS-II [35]	0.521	0.490	0.342	0.553	0.534	0.379
BMPRI [86]	*0.692*	0.583	0.422	0.555	0.534	0.382
BRISQUE [36]	0.565	0.411	0.289	0.426	0.398	0.276
CurveletQA [37]	0.560	0.471	0.337	0.471	0.442	0.316
DIIVINE [38]	0.521	0.487	0.340	0.429	0.436	0.307
ENIQA [87]	0.596	0.545	0.385	0.637	*0.641*	*0.466*
GRAD-LOG-CP [69]	0.662	0.627	0.454	0.590	0.570	0.415
GWH-GLBP [70]	0.315	0.357	0.245	0.302	0.285	0.196
IL-NIQE [27]	0.516	0.456	0.317	0.588	0.630	0.453
NBIQA [88]	0.695	*0.628*	*0.459*	*0.646*	0.615	0.446
NIQE [26]	0.263	0.277	0.184	0.302	0.338	0.228
OG-IQA [71]	0.564	0.452	0.321	0.527	0.447	0.314
PIQE [89]	0.491	0.364	0.255	0.289	0.237	0.201
Robust BRISQUE [90]	0.487	0.315	0.218	0.375	0.301	0.209
SSEQ [91]	0.615	0.520	0.373	0.454	0.434	0.304
LFD-IQA-SVR	0.637	0.645	0.470	0.845	0.838	0.640
LFD-IQA-GPR	**0.705**	**0.669**	**0.492**	**0.857**	**0.848**	**0.654**

**Table 5:** Direct and weighted average of PLCC, SROCC, and KROCC performance metrics. The best results are typed in bold, the second best results are underlined, and the third best results are typed in italic.

	Direct Average			Weighted Average		
**Method**	**PLCC**	**SROCC**	**KROCC**	**PLCC**	**SROCC**	**KROCC**
BIQI [85]	0.552	0.503	0.356	0.584	0.556	0.398
BLIINDS-II [35]	0.559	0.543	0.382	0.592	0.583	0.416
BMPRI [86]	0.633	0.591	0.413	0.647	0.623	0.434
BRISQUE [36]	0.590	0.541	0.384	0.615	0.582	0.417
CurveletQA [37]	0.638	0.605	0.414	0.658	0.633	0.431
DIIVINE [38]	0.610	0.590	0.407	0.631	0.618	0.424
ENIQA [87]	0.681	0.660	0.475	0.723	*0.711*	*0.521*
GRAD-LOG-CP [69]	0.670	0.656	0.465	0.691	0.678	0.491
GWH-GLBP [70]	0.545	0.539	0.377	0.588	0.578	0.403
IL-NIQE [27]	0.486	0.459	0.331	0.476	0.468	0.345
NBIQA [88]	*0.709*	*0.678*	*0.477*	*0.734*	0.710	0.495
NIQE [26]	0.295	0.325	0.218	0.292	0.340	0.229
OG-IQA [71]	0.603	0.553	0.408	0.629	0.590	0.442
PIQE [89]	0.274	0.222	0.160	0.255	0.221	0.159
Robust BRISQUE [90]	0.567	0.500	0.352	0.600	0.547	0.389
SSEQ [91]	0.578	0.541	0.392	0.598	0.577	0.421
LFD-IQA-SVR	0.758	0.749	0.554	0.807	0.799	0.596
LFD-IQA-GPR	**0.786**	**0.765**	**0.573**	**0.827**	**0.811**	**0.616**

**Table 6:** Results of the two-sided Wilcoxon rank sum test. Symbol ‘1’ is used to denote that the proposed method—LFD-IQA-SVR—is significantly better than the method in the row on the database in the column.

Method	CLIVE [21]	KonIQ-10k [22]	SPAQ [23]	TID2013 [24]	KADID-10k [25]
BIQI [85]	1	1	1	1	1
BLIINDS-II [35]	1	1	1	1	1
BMPRI [86]	1	1	1	1	1
BRISQUE [36]	1	1	1	1	1
CurveletQA [37]	1	1	1	1	1
DIIVINE [38]	1	1	1	1	1
ENIQA [87]	1	1	1	1	1
GRAD-LOG-CP [69]	1	1	1	1	1
GWH-GLBP [70]	1	1	1	1	1
NBIQA [88]	1	1	1	1	1
OG-IQA [71]	1	1	1	1	1
Robust BRISQUE [90]	1	1	1	1	1
SSEQ [91]	1	1	1	1	1

**Table 7:** Results of the two-sided Wilcoxon rank sum test. Symbol ‘1’ is used to denote that the proposed method—LFD-IQA-GPR—is significantly better than the method in the row on the database in the column.

Method	CLIVE [21]	KonIQ-10k [22]	SPAQ [23]	TID2013 [24]	KADID-10k [25]
BIQI [85]	1	1	1	1	1
BLIINDS-II [35]	1	1	1	1	1
BMPRI [86]	1	1	1	1	1
BRISQUE [36]	1	1	1	1	1
CurveletQA [37]	1	1	1	1	1
DIIVINE [38]	1	1	1	1	1
ENIQA [87]	1	1	1	1	1
GRAD-LOG-CP [69]	1	1	1	1	1
GWH-GLBP [70]	1	1	1	1	1
NBIQA [88]	1	1	1	1	1
OG-IQA [71]	1	1	1	1	1
Robust BRISQUE [90]	1	1	1	1	1
SSEQ [91]	1	1	1	1	1

**Table 8:** Results of the cross database test. The examined and the proposed methods were trained on KonIQ-10k [22] and tested on CLIVE [21]. The best results are typed in bold, the second best results are underlined, and the third best results are typed in italic.

Method	PLCC	SROCC	KROCC
BIQI [85]	0.477	0.424	0.289
BLIINDS-II [35]	0.107	0.090	0.063
BMPRI [86]	0.453	0.389	0.298
BRISQUE [36]	0.509	0.460	0.310
CurveletQA [37]	0.496	0.505	*0.347*
DIIVINE [38]	0.479	0.434	0.299
ENIQA [87]	0.428	0.386	0.272
GRAD-LOG-CP [69]	0.427	0.384	0.261
GWH-GLBP [70]	0.480	0.479	0.328
NBIQA [88]	0.503	*0.509*	0.284
OG-IQA [71]	0.442	0.427	0.289
Robust BRISQUE [90]	*0.516*	0.481	0.327
SSEQ [91]	0.270	0.256	0.170
LFD-IQA-SVR	0.567	0.561	0.390
LFD-IQA-GPR	**0.603**	**0.585**	**0.409**

## Data Availability

In this paper, the following publicly available benchmark databases were used: 1. CLIVE: https://live.ece.utexas.edu/research/ChallengeDB/index.html (accessed on 16 April 2022); 2. KonIQ-10k: http://database.mmsp-kn.de/koniq-10k-database.html (accessed on 16 April 2022); 3. SPAQ: https://github.com/h4nwei/SPAQ (accessed on 16 April 2022); 4. KADID-10k: http://database.mmsp-kn.de/kadid-10k-database.html (accessed on 16 April 2022); 5. TID2013: https://www.ponomarenko.info/tid2013.htm (accessed on 16 April 2022). The source code of the proposed LFD-IQA no-reference image quality assessment method is available at: https://github.com/Skythianos/LFD-IQA (accessed on 1 September 2022).

## Abbreviations

The following abbreviations are used in this manuscript:

BRIEF	binary robust independent elementary features
BRISK	binary robust invariant scalable keypoints
CNN	convolutional neural network
DCT	discrete cosine transform
FAST	features from accelerated segment test
FR	full-reference
GPR	Gaussian process regressor
GPU	graphical processing unit
HVS	human visual system
IQA	image quality assessment
KADID	Konstanz artificially distorted image quality database
KROCC	Kendall rank order correlation coefficient
LIVE	laboratory for image and video engineering
NR	no-reference
NSS	natural scene statistics
ORB	oriented FAST and rotated BRIEF
PLCC	Pearson linear correlation coefficient
RR	reduced-reference
SPAQ	smartphone photography attribute and quality
SROCC	Spearman rank order correlation coefficient
SURF	speeded up robust features
SVR	support vector regressor
TID	Tampere image database
